# Experimental Protocols to Test Aortic Soft Tissues: A Systematic Review

**DOI:** 10.3390/bioengineering11080745

**Published:** 2024-07-23

**Authors:** Rodrigo Valente, André Mourato, José Xavier, Pedro Sousa, Tiago Domingues, Paulo Tavares, Stéphane Avril, António Tomás, José Fragata

**Affiliations:** 1 UNIDEMI, Department of Mechanical and Industrial Engineering, NOVA School of Science and Technology, Universidade NOVA de Lisboa, 2829-516 Caparica, Portugal; rb.valente@campus.fct.unl.pt (R.V.); af.mourato@campus.fct.unl.pt (A.M.); 2 Intelligent Systems Associate Laboratory, LASI, 4800-058 Guimarães, Portugal; 3 INEGI, Faculty of Engineering, University of Porto, Rua Dr. Roberto Frias, 4200-465 Porto, Portugal; psousa@inegi.up.pt (P.S.); ptavares@inegi.up.pt (P.T.); 4 Mines Saint-Etienne, University of Lyon, Inserm, Sainbiose U1059, Campus Santé Innovation, 10, rue de la Marandière, 42270 Saint-Priest-en-Jarez, France; avril@emse.fr; 5 Department of Cardiothoracic Surgery, Santa Marta Hospital, Rua de Santa Marta, 1169-024 Lisboa, Portugal; acruztomas@gmail.com (A.T.); jose.fragata@nms.unl.pt (J.F.); 6 Department of Surgery and Human Morphology, NOVA Medical School, Universidade NOVA de Lisboa, Campo Mártires da Pátria, 1169-056 Lisboa, Portugal

**Keywords:** aorta, biaxial tests, digital image correlation, experimental protocols, hyperelastic behaviour, soft tissue

## Abstract

Experimental protocols are fundamental for quantifying the mechanical behaviour of soft tissue. These data are crucial for advancing the understanding of soft tissue mechanics, developing and calibrating constitutive models, and informing the development of more accurate and predictive computational simulations and artificial intelligence tools. This paper offers a comprehensive review of experimental tests conducted on soft aortic tissues, employing the Preferred Reporting Items for Systematic Reviews and Meta-Analyses (PRISMA) methodology, based on the Scopus, Web of Science, IEEE, Google Scholar and PubMed databases. This study includes a detailed overview of the test method protocols, providing insights into practical methodologies, specimen preparation and full-field measurements. The review also briefly discusses the post-processing methods applied to extract material parameters from experimental data. In particular, the results are analysed and discussed providing representative domains of stress–strain curves for both uniaxial and biaxial tests on human aortic tissue.

## 1. Introduction

The aorta, being the largest artery in the human body, plays a crucial role in distributing oxygenated blood and nutrients to different organs and tissues [1]. It is subject to dynamic mechanical forces due to the pulsating blood flow imposed by the heart [2]. These forces subject the aortic tissue to cyclic stretching, compression and shearing stresses throughout the cardiac cycle [3,4]. The ability of the aorta to accommodate mechanical loads while maintaining its structural integrity is critical for maintaining homeostasis. The aorta is a dynamic and complex tissue with unique structural and mechanical properties. It is composed of three layers: the intima, the media and the adventitia [5,6,7]. The tunica intima, the innermost layer, is a thin endothelial lining that provides a smooth surface for blood flow and is responsible for triggering the biochemical signal cascades. The tunica media, consisting mainly of elastin fibres and smooth muscle cells, contributes to the aorta’s ability to stretch and recoil. The outermost layer, the tunica adventitia, comprises collagen fibres and fibroblasts, providing structural support and anchorage to surrounding tissues.

Over the years, numerous experimental protocols have been developed to investigate the mechanical behaviour [8,9,10], composition [11,12,13], and microstructural [14,15] features of aortic tissue. Understanding the complex mechanical behaviour of aortic tissue is of paramount importance, elucidating the contributing factors to aortic pathology, such as aortic aneurysms, dissections and atherosclerosis, and guiding the development of therapeutic strategies [16,17,18]. Alterations in the mechanical properties of the aorta are associated with various pathological conditions. Understanding the underlying mechanisms of these diseases requires a comprehensive analysis of the mechanical behaviour and structural changes in aortic tissues. Also, characterising the mechanical properties of the aorta can aid in the development of improved diagnostic techniques and treatment strategies, in particular, by feeding robust computational frameworks with in vivo data [19,20]. For instance, accurate assessment of aortic stiffness can assist in risk stratification and guidance to therapeutic interventions. Furthermore, the mechanical properties of aortic tissues influence the design and performance of medical devices, such as stents and grafts, used in aortic surgeries.

Despite the importance of studying aortic soft tissues, several challenges exist in testing and evaluating their mechanical behaviour. Firstly, the aorta is a highly heterogeneous tissue, exhibiting regional variations in its composition and mechanical properties. This heterogeneity requires careful consideration of sample location and dimensions when interpreting experimental results. Additionally, the anisotropic nature of aortic tissues, characterised by direction-dependent mechanical properties, poses challenges in accurately assessing their behaviour under various loading conditions. Furthermore, the delicate balance between tissue preservation and experimental manipulation presents challenges in preserving the native mechanical properties of aortic tissues during testing. Factors including tissue preparation, storage and handling can alter the mechanical properties of the tissue, potentially leading to artefacts in the experimental results.

In this article, a systematic review following the Preferred Reporting Items for Systematic Reviews and Meta-Analyses (PRISMA) [21] methodology is presented, concerning the diversity of experimental protocols and post-processing methods applied to study the mechanical behaviour of vascular tissue, with a focus on aortic tissue. The objective is to answer the following scientific questions: “Which experimental protocols can be applied to mechanically test vascular tissue?” and “Which post-processing methods are used to extract material parameters from the experimental data?”. This paper is split into five sections: Section 2 explains how the search, selection and screening processes were performed following the PRISMA methodology. In Section 3, a summary of the selected articles is presented, and in Section 4, the raised questions are discussed. Finally, in Section 5, a summary of the review is given as final remarks.

## 2. Materials and Methods

### 2.1. Search Strategy

The Scopus, Web of Science, IEEE, Google Scholar and PubMed databases were chosen as resources to filter out relevant articles for this review, as they are each well known and internationally recognised [22]. This comprehensive search was updated until 1 May 2024, using the search expression: (“mechanical testing” or “constitutive” or “full-field measurements”) and (“biaxial” or “tensile” or “shear” or “inflation”) and (“vascular tissue” or “aorta” or “aortic”). Conference papers or proceedings, book chapters, and short surveys were not included in the preliminary results and no restrictions regarding date of publication and language were imposed.

### 2.2. Inclusion and Exclusion Selection Criteria

This search included studies that conducted mechanical tests on human or animal vascular tissue. Additionally, works on the mechanical testing of pathological vascular tissue and the extraction of material parameters from experimental data were also considered. The following criteria were used to exclude studies from the full-text review: (i) mechanical testing of non-arterial tissue (e.g., myocardia, valve leaflets, esophagus); (ii) development and testing of engineered tissue (e.g., biomaterials, hydrogels, addictive manufacture products); (iii) purely numerical studies that used experimental data for validation purposes; (iv) mechanical testing on external support devices (e.g., grafts, scaffolds, artificial valves); (v) assessment of the impact of drugs or treatments on tissue; (vi) only in vivo data; (vii) not within the scope of this review.

### 2.3. Study Selection

The screening process of the selected results was performed in two different stages. In the first stage, the title and abstract of all the research works were analysed, and only those that met the selection criteria were selected for full-text revision. In the second stage, the compatibility of each work with the inclusion and exclusion criteria was rechecked during full-text analysis. Additionally, before reading, analysis spreadsheets were developed to standardise and synthesise the data extraction process.

## 3. Results

### 3.1. Publication Overview

**Figure 1:**
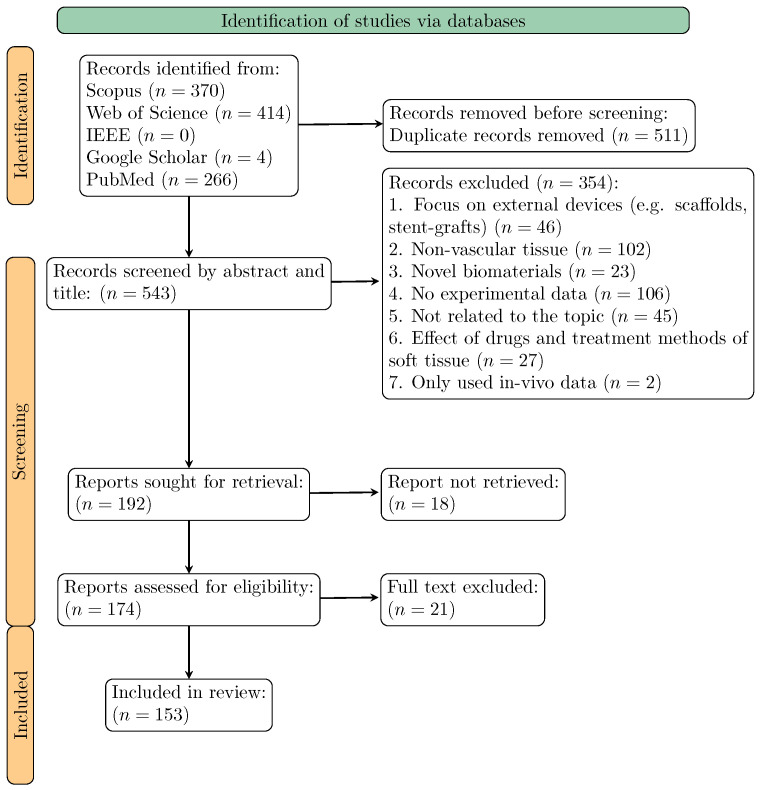
PRISMA flowchart of the systematic search strategy.

The initial search on the Scopus, Web of Science, IEEE, Google Scholar and PubMed platforms retrieved 1054 articles, which were reduced to 192 after eliminating duplicates and conducting the initial screening process. During full-text analysis, 21 articles were excluded as they did not meet the inclusion criteria. The details of the complete screening process are displayed in the PRISMA flowchart presented in Figure 1. The complete database after the second screening stage contained a total of 153 articles regarding the mechanical tests on vascular tissue and the subsequent post-processing of the experimental data. It is worth mentioning that six out of the 153 articles were also review papers. These reviews aimed to: (i) highlight the biomechanical environment of human arterial tissue and its unique mechanical response, and conduct a comprehensive review of the mechanical tests used to assess vascular tissue [23,24,25]; (ii) revisit the different techniques for exploring the role of elastin and collagen on arterial mechanical behaviour [26]; (iii) compare the data from uniaxial tensile tests on abdominal aorta aneurysms [27].

**Figure 2:**
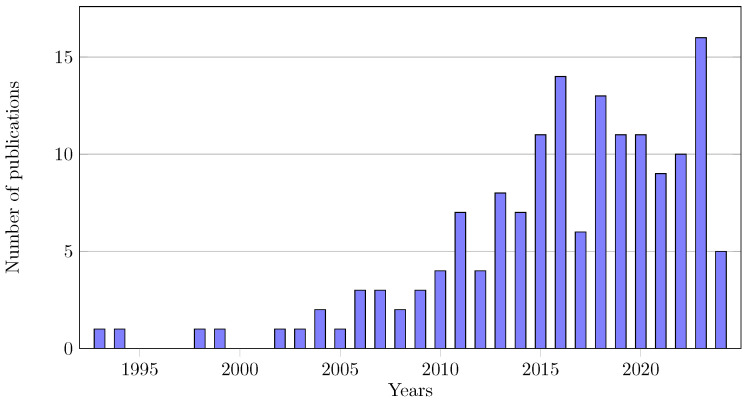
Evolution of the number of publications over recent years.

The current review aims to comprehensively analyse experimental protocols and post-processing methods used in the mechanical assessment of vascular tissue, namely, aortic tissue. It synthesises general practices, mechanical tests and extraction of material properties governing constitutive models. By providing insights into the methodologies employed for extracting material properties, it is meant to serve as a valuable resource for researchers and practitioners in the field. Despite the relevance of mechanical testing of soft tissues, unresolved questions still hinder the development of standardised guidelines. Figure 2 emphasises the increasing interest in this field over the past decade, underscoring the importance of exploring soft tissue mechanics to enhance our understanding of tissue behaviour and ultimately elucidate the factors contributing to the development and progression of pathologies, such as aneurysms.

**Figure 3:**
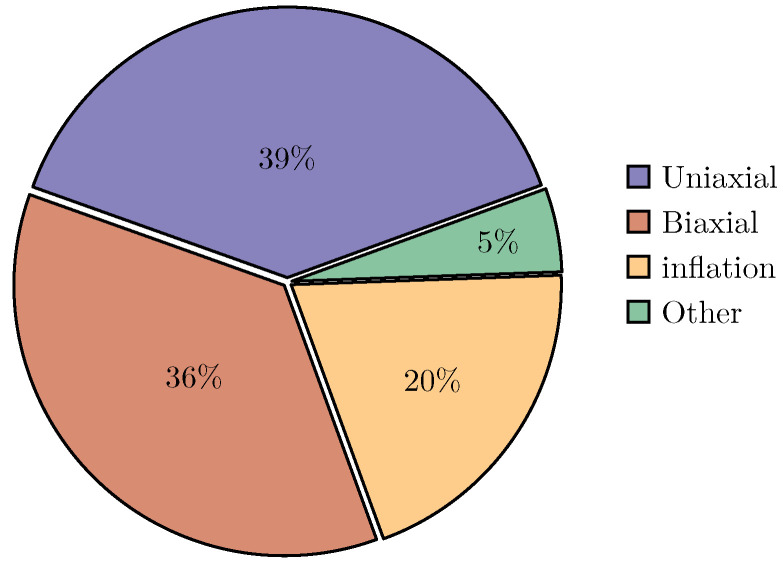
Relative frequence of the most commonly used mechanical tests on vascular tissue.

Figure 3 illustrates the distribution of mechanical tests employed in the selected articles. Among them, the most prevalent was the uniaxial tensile test, conducted in over a third (39%) of the analysed articles, possibly owing to its relative simplicity compared to counterpart tests. The second most commonly used mechanical test was the biaxial tensile test (35%), followed by inflation tests, with and without extension (21%).

**Figure 4:**
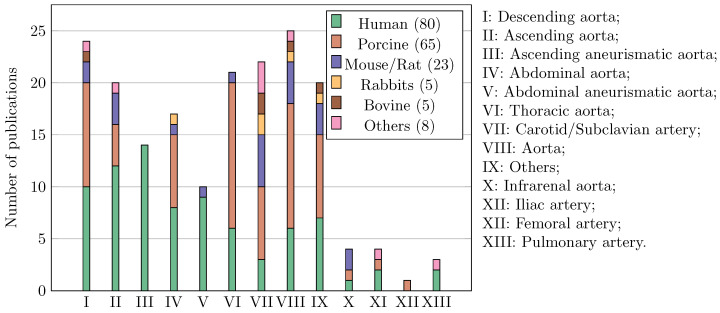
Type of tissue and anatomical region which the tissue was extracted from.

Another noteworthy aspect is the analysis of the types of tissue tested and their anatomical origins, as summarised in Figure 4. The findings reveal that human tissue was the most studied, despite ethical concerns surrounding its harvesting and testing. Porcine tissue emerged as the second most commonly studied tissue, owing to its similarities to human tissue. Regarding the anatomical region of tissue extraction, the majority were sourced from various sections of the aorta, including thoracic and abdominal regions and the sinotubular junction, and their branches, e.g., iliac, subclavian.

### 3.2. General Practices

Transversal practices were emphasised throughout the analysis, regardless of the specific mechanical test method. These practices encompass tissue preservation, bath conditioning during testing, and contactless full-field deformation measurements.

#### 3.2.1. Conservation Methods

Preserving organic materials for mechanical testing is crucial. The optimal approach would be to maintain fresh specimens with the utmost fidelity. However, logistical constraints, particularly with human tissues, require careful consideration of conservation techniques. This discussion aims to elucidate preservation strategies sourced from the scholarly literature and establish sustainable testing practices that mitigate environmental impact and tissue degradation.

Mechanical tests performed on fresh samples or within 6 h of harvesting are expected to yield the most reliable results [28,29,30,31,32,33,34,35,36,37,38,39,40,41,42,43,44,45,46,47]. Different procedures have been reported in the literature to maintain tissue integrity between harvesting and testing, including keeping the tissue heated at physiological temperature and hydration [48,49,50], or conditioning it at lower temperatures [51,52,53,54,55]. However, there is a lack of evidence to determine which method is most effective for preserving tissue integrity. Studies have been conducted to infer how preservation conditions and time will affect the tissue microstructure and, therefore, the mechanical properties. Some studies have preserved the samples at a temperature around 4 °C for small-time frames (within 48 h) [9,56,57,58,59,60,61,62,63,64,65,66,67,68,69,70,71,72,73,74,75,76,77,78], whilst other protocols have frozen samples (−20 °C or even −80 °C) for longer periods [3,5,8,11,45,46,47,77,79,80,81,82,83,84,85,86,87,88,89,90,91,92,93,94,95]. Furthermore, the most common solutions for submerging the tissue under those periods are Physiologic Saline Solution (PSS) and Phosphate-Buffered Saline (PBS). From these studies, it is possible to infer that testing fresh and frozen tissues has no significant impact on the mechanical properties.

PSS is a 0.9% solution of sodium chloride and is so-called “physiological” because it is an isotonic solution with an osmotic pressure equivalent to that of body fluids, such as blood, under normal conditions with a pH of 7.4. Cosentino et al. [96] used a calcium and glucose free PSS to preserve the samples at −80 °C for preservation, although other options seem more common for these temperatures. A few authors only refer to the solution as a saline solution with no further details [14,69,97,98,99,100,101,102,103,104,105,106].

PBS is a buffer solution with a pH of around 7.4. Although both PBS and PSS are isotonic and have similar pH levels, they differ in composition and usage. PBS is a more complex solution containing additional buffering agents, which makes it more suitable for maintaining the pH and osmotic balance of cells in laboratory experiments. PBS is commonly used in cell culture, immunohistochemistry and molecular biology experiments to maintain the pH and osmotic balance of cells and tissues. Some authors prefer to use it to preserve the samples at 4 °C for small conservation times [107,108,109,110,111,112,113,114,115,116,117] and at −20 °C [118,119]. In comparison to PSS, PBS is utilised more to preserve specimens for the long term at −80 °C [120,121,122,123,124].

A variant of PBS known as Dulbecco’s Phosphate-Buffered Saline (DPBS) has also been mentioned and introduced. DPBS is commonly used in cell culture experiments to maintain the pH (between 7.2 and 7.6) and osmotic balance of cells and tissues [125]. Including additional components in DPBS, such as calcium and magnesium ions, generates a more effective buffer solution for sustaining the viability and functionality of cultured cells compared to PBS alone.

Krebs–Ringer Solution (KRS) is another commonly used solution in laboratory settings. Compared to PBS and PSS, KRS contains additional ions, such as calcium and bicarbonate, making it a more versatile and physiologically relevant solution for maintaining the function of isolated organs, tissues and cells. Moreover, KRS has been employed in medical settings to sustain the viability of donated organs prior to transplantation. This solution was used in a few studies to conserve samples at 4 °C before testing [126,127].

Dulbecco’s Modified Eagle Medium (dMEM) is a medium used to support the growth of mammalian cells. It comprises a mixture of nutrients and vitamins necessary to meet the requirements of cells. Niestrawska et al. [12] used it at 4 °C to preserve the samples until testing, while Geelhoed et al. [128] employed it for transportation before freezing the samples at −80 °C.

Additionally, Cavinato et al. [32] utilised Hank’s Balanced Salt Solution (HBSS) to preserve the samples at 4 °C. HBSS is a cell culture medium for various cell types, including primary and established cell lines. It finds applications in other biological procedures, such as tissue dissociation, cell washing and resuspension, and sample preparation for analysis. HBSS can be supplemented with additional components, such as antibiotics, growth factors and serum to promote cell growth and function.

#### 3.2.2. Bath during Test

Mechanical testing on soft tissue is typically conducted using a temperature-controlled bath, offering the advantage of replicating ex vivo conditions that mimic the in vivo physiological environment, thereby preserving the inherent properties of the tissue. This approach effectively mitigates external perturbations, such as temperature fluctuations or specimen dissection. Some studies have reported a protocol using an ultrasonic humidifier to maintain humidity of the tissue during the mechanical test [61,92,94], while others have opted to spray a saline solution [42,58,61,73,90,92,106,129,130,131] or to brush it [65]. Strategically, using spray solutions can be cost-effective and eco-friendly by reducing solution volumes. Nevertheless, spray applications may suffer from the limitation of inconsistent sample coverage.

The bath can be filled with different fluids to simulate physiological conditions. The primary fluids used are PBS [4,15,30,34,39,45,47,48,51,53,57,67,83,84,86,87,93,94,96,101,108,109,112,113,115,117,119,120,121,128,132,133,134,135,136,137,138,139,140,141], DPBS [28,142] and PSS [9,54,55,63,66,91,95,123,124,143,144,145]. Some authors note the use of a saline solution; it can be assumed they are referring to a physiological solution, although this is not guaranteed [12,14,31,36,50,62,68,69,79,80,81,82,111,125,146,147].

Krebs–Henseleit Solution (KHS), Ringer Solution (RS) and KRS have also been employed as baths in mechanical tests of arterial tissue [33,35,37,41,60,104,126,127,148,149,150]. These solutions play a crucial role in maintaining the tissue in a physiological state by providing the necessary ions and nutrients. KHS is typically used for experiments involving isolated tissue, RS is commonly used for short-term experiments, and KRS has been applied in both scenarios. In the literature, references to using HBSS [40,151,152,153] and dMEM [13] in mouse/mice tissue are reported. However, these solutions possess similar properties and fulfil the same role as the previously mentioned ones. Lastly, although not an isotonic solution, some authors have used water as the immersion bath [52,70,71,99,100,102,154].

#### 3.2.3. Full-Field Measurements

A fundamental issue regarding the mechanical testing of soft tissues is the ability to accurately measure tissue deformation under loading. This is particularly important for characterising the mechanical properties and validating computational models. Several techniques have been employed to measure the deformation of soft tissues during mechanical tests, including Digital Image Correlation (DIC) and image-based tracking techniques, or use of a video-extensometer. These techniques are contactless and offer high-resolution, potentially enabling the characterisation of regional variations in mechanical deformation and properties, as well as the assessment of the tissue’s response to different loading conditions. The choice of a given image-based technique depends on the specific requirements of the experiment, such as the desired spatial resolution, measurement accuracy and the tissue’s response time.

DIC is particularly useful for studying the deformation of complex materials as it can capture the heterogeneous deformation across an entire region of interest [43,46,47,56,69,85,91,92,93,94,102,106,113,117,130,133,152,155,156,157]. However, it requires a transfer of a suitable speckle pattern to the surface of interest [158], and the accuracy of DIC measurements is strongly related to factors such as the quality of the speckle pattern, setting parameters and the spatial resolution of the cameras [159,160]. Challenges in speckle pattern creation include ensuring pattern quality while maintaining specimen hydration, pattern adherence during large deformations, and addressing refractive index changes when imaging submerged samples. An effective procedure for creating speckle patterns on biological soft tissue involves staining the tissue with methylene blue solution to achieve a dark background and airbrushing the surface with paint to create a contrasted speckle [158,161]. These methods offer reliable and effective ways to create speckle patterns for accurate strain measurements in biological soft tissues.

Image-based tracking techniques may be more easily applied, relying on positioning a few markers on the tissue surface. However, this comes at the cost of losing spatial resolution and local strain measurements [8,13,15,28,51,57,61,62,63,64,65,68,73,76,99,104,114,116,123,124,125,126,139,145,147,162,163]. The most common methods to mark the sample for this technique are beads [37,100,112,164], black powder (normally carbon or graphite) [29,66,86,96,140] and ink [36,45,47,52,95].

Other techniques have also been developed at different scales of analysis. In situ characterisation of the mechanical behaviour at the microscopic level has been carried out to study the relationships between microstructure and mechanical properties [70,154], as well as used to train advanced deep learning algorithms to quantify the individual contributions of various microstructural features to the macroscopic mechanical properties [40]. Optical Coherence Tomography (OCT) has also been developed. This technique uses light interference to capture cross-sectional images of biological tissue microstructure, creating a three-dimensional body. It can be used to visualise the internal microstructure of the tissue and pinpoint changes in its shape during deformation [151]. OCT can provide valuable insights into the mechanical behaviour of aortic tissue and its response to loading conditions. Acosta-Santamaría et al. [165] applied Digital Volume Correlation (DVC) with OCT imaging to track the deformation over time in vascular tissue, providing initial insights into the three-dimensional temporal deformation of microstructure [84]. Optical or laser extensometers are a precise technology used to measure distances [39,58,101]. Piezoelectric sensors can directly measure tissue deformation [60], while ultrasound imaging can provide detailed real-time images of the tissue and its deformation [68,83,98]. Supersonic shear wave elastography was used by Shcherbakova et al. [42] as a non-invasive technique to measure tissue mechanical properties by generating supersonic shear waves and measuring their propagation through the tissue. In a mechanical test, it was used to find out the tissue’s shear modulus during deformation, which is a key part of understanding its mechanical characteristics.

### 3.3. Mechanical Test Methods

#### 3.3.1. Uniaxial Test

The proposed fixation of cardiovascular tissue in a uniaxial test can be challenging because the specimen needs to be maintained at a specific level of hydration. The most common method to overcome this type of limitation is the usage of clamps [30,39,42,50,60,71,75,79,90,94,100,101,106,117,118,129,131,149]. To enhance the grip between the clamps and the tissue, some researchers have found that using personalised serrated clamps [5,73,136,144] or clamps lined with sandpaper [31,35,59,70,92,105,127,164] yields adequate results. Other authors have even proposed directly gluing the tissue to the clamps, eliminating any possible slippage [3,28,43,50,58,85,92,141].

Mechanical testing of aortic tissue using uniaxial loading has been carried out using diverse preconditioning protocols. The purpose of applying pre-stretching cycles to the tissue is to achieve a consistent and reproducible mechanical response representative of the in vivo conditions, ensuring reliable data. The standard procedure for prestressing involves subjecting the specimen to a specified number of load cycles, typically ranging from two to twenty, with the majority opting for ten cycles. The preconditioning speed is normally slow, at a quasi-static velocity of 0.01 to 0.1 mm s^−1^ to prevent tissue damage [39,47,65,90,106,129,131,136,144,146]. The strain levels applied during preconditioning typically range from 10% to 40% [3,31,38,39,43,50,61,65,74,80,97,106,118,129,131,136,138,144,146].

The uniaxial test is conducted following the preconditioning loading stage, during which the final cycle is typically carried out until rupture occurs [14,47,49,50,51,61,71,82,105,106,117,118,129,131,136,144,146,149,164]. In some studies, however, the uniaxial tests are performed up to a predefined loading threshold to prevent damage, thereby preserving the tissue for subsequent histological analysis [35,42,73,97,130,166]. The large majority of authors decide to perform a quasi-static experiment with the same velocity as preconditioning. Polzer and Vitásek [79] investigated the low cycle fatigue of porcine aortic samples tested in a circumferential direction, with a focus on cyclic loading and static strength comparison. Other studies addressed relaxation tests to characterise the viscoelastic properties of vascular tissue [43,58,75,84].

#### 3.3.2. Biaxial Test

The biaxial test is a valuable method for understanding the mechanical behaviour of arterial tissue [167]. In this test method, the sample can be loaded in two orthogonal directions, providing a more enlightening understanding of the anisotropic mechanical response. Therefore, this method enables the evaluation of tissue responses under various stress states and loading conditions. Typically, the device uses linear carriages to ensure symmetric biaxial deformation of the specimen, and it is equipped with pairs of load cells and a fixation system for the tissue. The specimen can be submerged in a physiological saline solution at a controlled temperature, and image-based techniques can be used for contactless deformation assessment. The independent control of displacements in both loading directions can enable the measurement of stress–strain data for various biaxial stress states.

**Figure 5:**
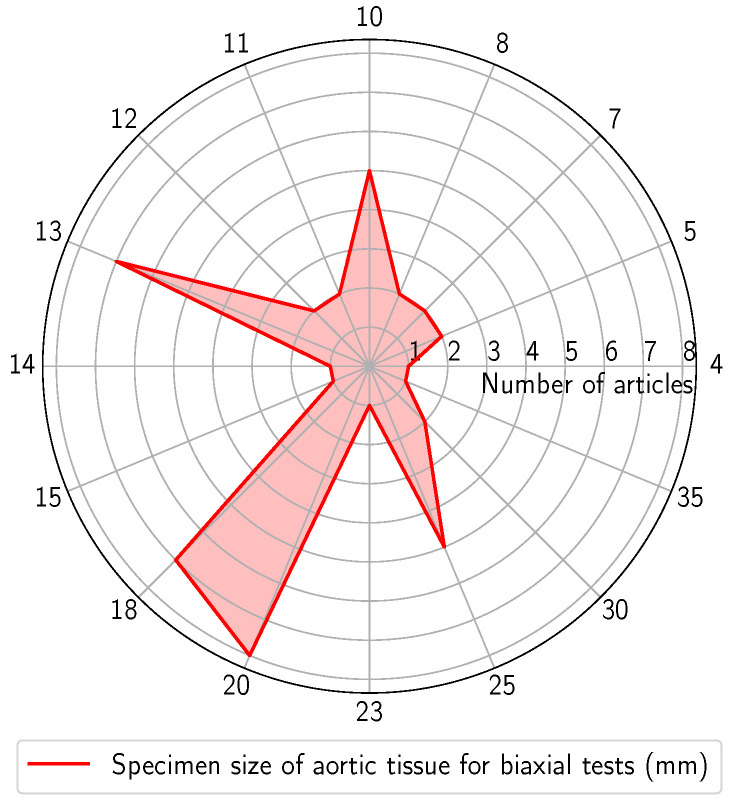
Typical sizes of rectangular specimens used in the biaxial tests (units: mm).

For biaxial tests, the specimens predominantly consist of square or cruciform geometries. These shapes allow for applying tensile loads along two axes simultaneously at independent velocities. Cruciform samples have the advantage of simple fixation systems, facilitating grasping and uniform load application. Additionally, there is a reduced probability of rupture near the clamp region [57,66,67,122]. However, cruciform samples are limited by the amount of material required and, therefore, are not usually suitable for arterial soft tissues. To tackle this issue, square-shaped specimens have been proposed. Figure 5 summarises the typical sizes of the square specimens used in the biaxial tests, presented as a function of the number of articles. As can be seen, specimens ranging from 4 to 35 mm have been employed, with a prevalence between 10 and 20 mm.

Similar to the uniaxial test protocol, biaxial testing devices also have certain limitations concerning the fixation of arterial soft tissue. Hydration and heated bath conditions can generate a slippery surface during the gripping process. Therefore, the common approach for fixing the tissue involves the use of hooks [12,36,54,55,64,67,72,77,83,89,96,114,116,120,121,123,124,125,132,134,145]. Some authors have opted for clamps as an alternative to prevent slippage [11,15,44,57,81,93,94,99,100,147]. Other researchers have employed rakes or surgical staples [4,109,143]. Other fixation methods have been reported to effectively prevent slippage and allow for sample reuse, such as sutures or adhesives [28,29,53,62,115,122,140,150,153].

The preconditioning cycling protocol closely reproduces that presented for the uniaxial test. The objective is to subject the sample to loading and unloading cycles, typically around 10 [15,44,55,66,72,80,83,93,95,99,108,111,121,124,125,132,134]. It is standard for both the preconditioning phase and the subsequent test protocol to start the test with a slight preload, typically below 0.1 N, to uphold tension on the tissue [15,80,120,124].

The general experimental protocol for the biaxial test involves loading using ratios between the axial and circumferential directions. These ratios typically include fractions such as 1:1, 0.75:1, 0.5:1, 0.25:1, which are repeated in both directions until a defined strain or stress level is achieved [4,12,29,45,48,57,62,64,69,77,78,86,88,94,109,111,114,115,116,123,132,140,168,169]. Nonetheless, the protocols exhibit variations in terms of test velocity and strain levels, despite the fact that most authors carry out the test in quasi-static conditions [55,89,108,120,122,143,145]. Biaxial tests have been performed at a controlled displacement rate between 0.05 and 0.35 mm s^−1^ [11,15,36,67,72,81,96,121]. Other authors have followed a procotol where samples were subjected to equibiaxial displacement control, in which deformations progressively increased to values in the range of 10% up to 35% with increments of 5% [66,99,100,125]. When performing biaxial tests, some authors have decided to continue testing until rupture [12,28,81]. Nevertheless, the prevalent procedure is to restrict the applied strain to a defined value, typically ranging between 15% and 60% of the initial length [15,45,95,111,125], or to limit the applied stress to approximately 15–60 kPa [44,62,77,88,93,132,140] or even 240 kPa [94].

In addition to the biaxial protocol, some authors have conducted an additional test to extract more information or to validate the protocol itself. O’Leary et al. [95], Kamenskiy et al. [54] and Jadidi et al. [34] performed a second 1:1 test after the initial protocol, using the same test conditions, to ensure that the tissue remained undamaged and to assess any potential effects on mechanical properties. Schroeder et al. [80] and Lu et al. [134] included a 0:1 test in both directions within the protocol to obtain direction-specific uniaxial data alongside the biaxial results. While this method cannot fully replace uniaxial testing, which can subject the tissue to higher strains without damage, it does provide complementary information regarding direction-specific deformation.

#### 3.3.3. Inflation Test

The inflation test involves subjecting a tubular specimen of arterial tissue to controlled internal pressure, mimicking physiological inflation conditions. By scrutinising how arteries respond to pressure loading, researchers gain a deeper understanding of biomechanical behaviour and refine the characterisation of anisotropic properties.

In the inflation test, the specimen is peripherally fixed, and the pressure is applied across the internal surface. Prior to testing, the majority of authors performed preconditioning cycles to ensure a consistent mechanical state [32,98,119,151]. The pressure applied during this procedure typically mirrors the experimental protocol (in vivo pressure), and although the number of cycles varies, the majority fall within 3 to 5 cycles [13,33,40,41,68,76,112,142,152,162]. The typical inflation protocol is defined with a predetermined pressure threshold, normally ranging between 0 and 200 mmHg [33,41,101,112,126,137,162,170] or, more commonly, under in vivo pressure conditions (80–120 mmHg) [9,13,32,37,68,76,87,119,133,135,139,142,151,152,154,171].

In addition to the internal pressure, some authors applied a fixed axial load to the sample during pressurisation to better simulate aortic prestretch [9,13,33,40,41,87,119,126,150,151,152,153,154,170,171]. Others opted to maintain a fixed pressure while varying the axial stretch [32,33,37,87,142]. Laffey et al. [87] defined a strain value of 40%, whilst other authors have defined a maximum stretch ranged between 1.05 and 1.85 [9,13,33,37,63,83,101,104,119,133,137,152,154]. Collins et al. [135], Collins et al. [142] and Eberth et al. [171] have defined a minimal force of 24.5 mN, 14.7 mN and 8.8 mN, respectively, while Schulze-Bauer et al. [170] applied a force of 5.9 N. Sáez et al. [63] performed a relaxation test with a defined stretch and an insufflation pressure of 300 mmHg; the data were extracted after 300 s.

#### 3.3.4. Other Tests

In addition to the previously mentioned mechanical test methods for soft tissue, some authors have proposed alternative setups. The bulge inflation test is a mechanical test using a rectangular or circular specimen held in a chamber between two plates and insufflated, creating a bulge from which the mechanical properties of the tissue can be evaluated [52,56,102,113]. The specimen sizes typically range between 30 mm × 30 mm and 45 mm × 45 mm and are clamped between the plates. The specimens undergo preconditioning cycles and are pressurised up to 75 mmHg [52,56,113]. During the experimental protocol, Dwivedi et al. [113] have defined a maximum pressure of 115 kPa increased in 12 steps and held for 5 s. This procedure was chosen to minimise the viscoelastic contributions for estimating the elastic properties. Davis et al. [102] inflated the sample by administering water into the inner cavity via a syringe pump operating at a rate of 2 mL min^−1^. Kim et al. [56] conducted an inflation test by pushing a cylinder piston at a constant speed of 15 mm min^−1^ until the aneurysmal tissue ruptured, while simultaneously measuring the applied pressure generated by supplying water to the back face of the tissue.

Sanders et al. [8] introduced a new insufflation technique, so-called ring-insufflation. The main focus was to apply pressure within working loads ranging from 0 to 120 mmHg to a ring with a thickness of 0.7 mm, secured between two parallel microscope slides. Deformation was recorded using a high-speed digital camera. The method was partially validated through successful testing with rubber rings and a vessel inflation test.

Sommer et al. [172] performed extension–inflation–torsion experiments on human subclavian and common iliac arteries to evaluate their biomechanical properties. Unlike planar biaxial tests, extension–inflation–torsion tests closely replicate the physiological loading conditions of arteries. The tests involved continuous recording of the axial force, transmural pressures, outer diameter, axial gauge length, twisting angle, and torque under physiological and supra-physiological conditions.

García-Herrera et al. [39] developed a mechanical test apparatus enabling bending, axial stretching and internal pressurisation of the sample in a quasi-static manner. The main objective was to evaluate arterial vessel and tissue behaviour under large deformations. The protocol consisted of initially removing the arch bending by 90 °, followed by a longitudinal stretch of 1.7 and a pressurisation of 200 mmHg.

### 3.4. Experimental Results and Post-Processing

As previously demonstrated, the experimental protocols vary across multiple studies reported in the literature. This variation inevitably contributes to the dispersion of results, making it essential to analyse the impact of each protocol on the experimental outcomes. Articles that utilised tissue from the human ascending and abdominal aorta without alteration or tissue separation were selected and analysed separately, as they are the most common. Articles without preconditioning were not considered, as the tissue preparation before the experimental test can significantly affect the results. The experimental protocol, together with patient data, including age, sex and diseases, was taken into account to profile the results. Although sample size may impact the results, this was not considered in the analysis due to a lack of data. The results were categorised into uniaxial and biaxial tests due to a lack of experimental data in humans in other experimental protocols. This section will present an overview of results of the selected works. Regional domains of mechanical response, presenting data qualitatively in terms of stress–strain curves, will be systematically included and discussed.

#### 3.4.1. Uniaxial Test

**Figure 6:**
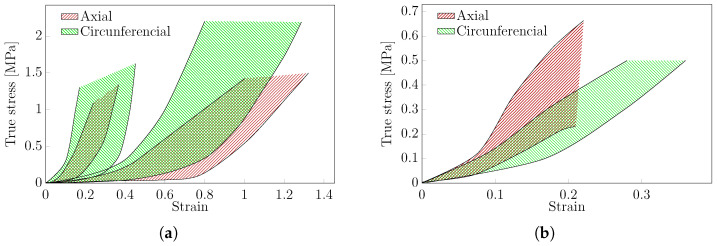
Representative stress–strain curves obtained from uniaxial tests on human tissue from: (**a**) thoracic aorta, (**b**) abdominal aorta.

Within the complete article database, only nine articles performed a uniaxial experimental analysis on the human aorta. These results were analysed separately and divided into thoracic and abdominal aorta. Figure 6 presents the stress–strain curves obtained from uniaxial tests on both (a) thoracic and (b) abdominal aorta, respectively. Additionally, the typical green and red shadow areas represent the uniaxial tests performed in the axial and circumferential directions, respectively. Concerning the thoracic aorta (Figure 6a), both axial and circumferential regions shifted to the right on the plot were extracted from [97,101,136], while those shifted to the left were reported in Li et al. [38,106]. The different stress–strain responses obtained among these studies may be caused by the fact that different experimental protocols were used. Nevertheless, both regions demonstrate the relevance of age on mechanical properties; an increase in patient age corresponds to an increase in stiffness attributed to elastin content decay [101,106]. Thus, ageing emerges as an important parameter to take into account in silico modelling. However, gender was not relevant in the results [101,106]. For the abdominal aorta (Figure 6b), four articles were selected based on the above criteria. It is noted that O’Leary et al. [90] and Martufi et al. [146] only presented results on either the axial or circumferential directions, respectively. Both the axial and circumferential regions were defined by the results extracted from patients with Abdominal Aorta Aneurysms (AAA) [14,90,146]. Only Kobielarz [14] included non-aneurysmal tissue in the uniaxial test, and the results are consistent with the presented areas. In both the thoracic and abdominal aorta, hyperelastic anisotropic behaviour was observed, as expected.

#### 3.4.2. Biaxial Test

**Figure 7:**
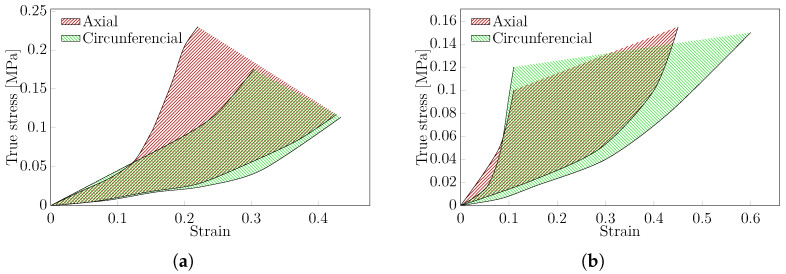
Representative stress–strain curves obtained from biaxial tests on human tissue from: (**a**) thoracic aorta, (**b**) abdominal aorta.

Based on the criteria described above, 11 articles focusing on thoracic aorta [4,44,69,86,89,96,109,114,123,143,145] together with five articles on the abdominal aorta, were selected [12,36,62,64,95]. The representative region for the stress–strain curves typically obtained in biaxial tests for human aortic tissue is shown in Figure 7. As noticed on the uniaxial test, age increase is correlated with a stiffer aorta [4,62,86,114,123]. Therefore, the results comparison cannot be performed due to the impact of the protocol on the experimental results. Furthermore, there are significant differences in results from articles with similar protocols demonstrating the importance of better standards for experimentally determining the mechanical behaviour of such tissues.

#### 3.4.3. Post-Processing

Relevant material parameters governing constitutive laws are usually derived from raw experimental data using a numerical fitting approach. Among various constitutive material laws, hyperelastic models are commonly employed to describe the behaviour of arterial tissue. This section briefly overviews and quantifies fitting methods used to extract relevant material properties.

Curve-fitting aims to adjust a mathematical model to fit the pattern of a set of data points, aiming to find the optimal parameters that minimise the difference between the model and the observed data. The least-square method has been extensively used to fit experimental data [4,5,39,48,97,101,119]. Due to the complexity of hyperelastic models, the Levenberg–Marquardt algorithm has been widely used in the literature to perform nonlinear least squares fitting [14,32,34,37,54,58,61,63,64,90,94,95,99,125,166,170]. Other general purpose optimisation algorithms, such as Nelder–Mead, have also been employed but tend to be less robust for complex hyperelastic models [41,52,89,93,126,140,143].

Several software programs can assist with this numerical task. For recording, standard tools include Hyperfit [11,36,80,120,121,136,144,145], HyperStudyTM [110], Maple [76], MicroCal Origin [35,59], SigmaStat [62], SPSS [106] and MATLAB [29,44,72,81,112,113,139,142,168], the former primarily using the Fmincon function [8,9,69,78,86,100,107,116,117,118,162] or the Isqnonlin function [12,28,67,96,124,135]. While MATLAB is renowned for its versatility and integration with various optimisation functions, other tools like Hyperfit are specialised for hyperelastic material fitting, offering more user-friendly interfaces for specific tasks. The finite element method (FEM) interactive approach or inverse methods have also been used successfully for this task [51,122,123,127].

Two primary types of hyperelastic models are employed to characterise the mechanical behaviour of arterial tissue: isotropic and anisotropic. The isotropic model describes the mechanical properties of the extracellular matrix within arterial tissue, while the anisotropic model considers the mechanical behaviour of the fibres. Typically, these materials are considered incompressible. The isotropic model simplifies data comparison as it depends on fewer variables. Standard isotropic models used by researchers include the Neo-Hookean model [41,109,111,119] and the Mooney–Rivlin model [100,124,173]. The mentioned models simplify data comparison by depending on fewer variables, making it easier to implement but potentially less accurate for capturing complex tissue behaviours.

These isotropic models are often integrated into anisotropic models, describing the tissue matrix alongside an anisotropic component of the Strain Energy Density Function (SEDF), which accounts for collagen and elastin fibres. The most prevalent anisotropic model for arterial tissue is the Holzapfel–Gasser–Ogden (HGO) model [3,5,8,9,14,29,32,40,48,57,61,65,66,72,73,80,81,89,93,94,101,102,107,108,109,113,122,123,131,144]. Additionally, the Fung model is employed to describe the anisotropic behaviours of tissue [35,37,41,45,47,59,62,92,106,112,114,121,125,126,143,154,164].

In summary, while isotropic models are the simplest approach, anisotropic constitutive laws, such as HGO, significantly improve model accuracy by considering fibre orientation. These models provide a better fit for the experimental data and are preferable for numerical simulations.

## 4. Discussion

The uniaxial mechanical test has been employed to collect experimental data on arterial tissue until rupture. Unlike other experimental protocols, the uniaxial test, along with the bulge test, consistently induced damage and rupture in the region of interest, thus enabling analysis of ultimate strength stresses.

While not the most common, the biaxial protocol is the test that reveals more significant anisotropic properties of the tissue. Moreover, biaxial tests have been primarily used to test human tissue. This is due to restrictions on specimen dimensions and the possibility of applying physiological deformation by controlling the applied force in the circumferential and axial directions.

The inflation test presents a particularly intriguing correlation between in vivo conditions and the mechanical properties of the tissue. Although it is not the most common test, the results are highly compelling and can offer a deeper understanding of tissue behaviour.

The typical stress–strain curves for both uniaxial (Figure 6) and biaxial (Figure 7) tests were estimated for human thoracic and abdominal aorta in both circumferential and axial directions. This region represents average behaviour, including dispersion, which can be induced by different experimental protocols. A few tests were excluded from these regions due to inconsistent results. There are not enough data on patients to understand the impact of the protocol on the results.

The presented review has certain limitations. The experimental data vary greatly within the protocols, leading to significant dispersion of results. This variability limits our ability to draw definitive conclusions about the impact of certain factors on experimental outcomes. For example, variations in sample size and test velocity can significantly influence the results. In that regard, the need for standardised protocols is strongly highlighted. Implementing such standards would not only improve our understanding of arterial mechanical behaviour but also help to validate both experimental and numerical results overall [174]. In this review, the focus was on the experimental protocol and general experimental true stress–strain curves. While a post-processing section is included, it only involved a qualitative analysis. Given its importance and complexity, future work should specifically address this aspect to comprehensively describe the various complex numerical models and fitting parameters found in the literature, and their influence on the results.

## 5. Conclusions

This review article employed the PRISMA methodology to select studies that addressed experimental protocols for testing aortic soft tissues. The main focus of the study was to analyse the most commonly used protocols in various experimental tests and their impact on outcomes. The studies predominantly concentrate on human and porcine aorta, specifically thoracic aortic tissue.

Tissue conservation can be categorised into three approaches: (i) Fresh, if the tissue is used immediately post-extraction; (ii) Refrigerated, if used within a few hours after extraction; and (iii) Frozen, if used after several days. Ideally, tissues should be tested fresh, although studies indicate frozen samples generally do not exhibit significant alterations. For preservation, tissues are typically stored in a PBS medium, which is also used to submerge the tissue during testing to replicate in vivo conditions.

The most commonly used tests reported in the literature are uniaxial, biaxial and inflation tests. In the uniaxial test, specimens are commonly dog-bone shaped to enhance fixation and prevent tissue rupture near the clamps. The biaxial protocol is more frequently used with human tissue, where the standard specimen shape is rectangular. However, there was some variability in specimen sizes in the biaxial protocol. In both tests, preconditioning is commonly employed, typically involving 10 cycles, and the testing velocity is maintained in a quasi-static manner throughout. The inflation protocol is conducted on tubular-shaped specimens under a fixed pressure, with or without an axial load. Preconditioning is also prominently featured in the reviewed protocols, and the primary objective of this mechanical test is to replicate in vivo conditions.

For experimental data in the uniaxial and biaxial testing protocols, we have delineated a representative mechanical response in the stress–strain curves for thoracic and abdominal aortic tissues. This designated region can be used for comparison and modelling purposes. The anisotropy of the tissue is demonstrated through variations within these regions in both the axial and circumferential directions. Patient age is a crucial parameter, as increases in tissue stiffness and significant reductions in ductility are observed with ageing. The reported sample size and precision were inadequate for detecting any significant changes attributable to variations in the experimental protocol.

## Data Availability

The original contributions presented in the study are included in the article, further inquiries can be directed to the corresponding author.

## Abbreviations

The following abbreviations are used in this manuscript:

AAA	Abdominal Aorta Aneurysms
DIC	Digital Image Correlation
dMEM	Dulbecco’s Modified Eagle Medium
dPBS	Dulbecco’s Phosphate-Buffered Saline
DVC	Digital Volume Correlation
HBSS	Hank’s Balanced Salt Solution
HGO	Holzapfel–Gasser–Ogden
KHS	Krebs–Henseleit Solution
KRS	Krebs–Ringer Solution
OCT	Optical Coherence Tomography
PBS	Phosphate-Buffered Saline
PRISMA	Preferred Reporting Items for Systematic Reviews and Meta-Analyses
PSS	Physiologic Saline Solution
RS	Ringer Solution
SEDF	Strain Energy Density Function
